# Supplementary material for: Hepatocytic expression of human sodium-taurocholate cotransporting polypeptide enables hepatitis B virus infection of macaques
Source: Nat Commun. 2017 Dec 15;8:2146. doi: 10.1038/s41467-017-01953-y (PMC5732258; doi:10.1038/s41467-017-01953-y)
Supplement: Supplementary file 1 — Supplementary Information [file 41467_2017_1953_MOESM1_ESM.pdf]

Supplementary Figure 1.

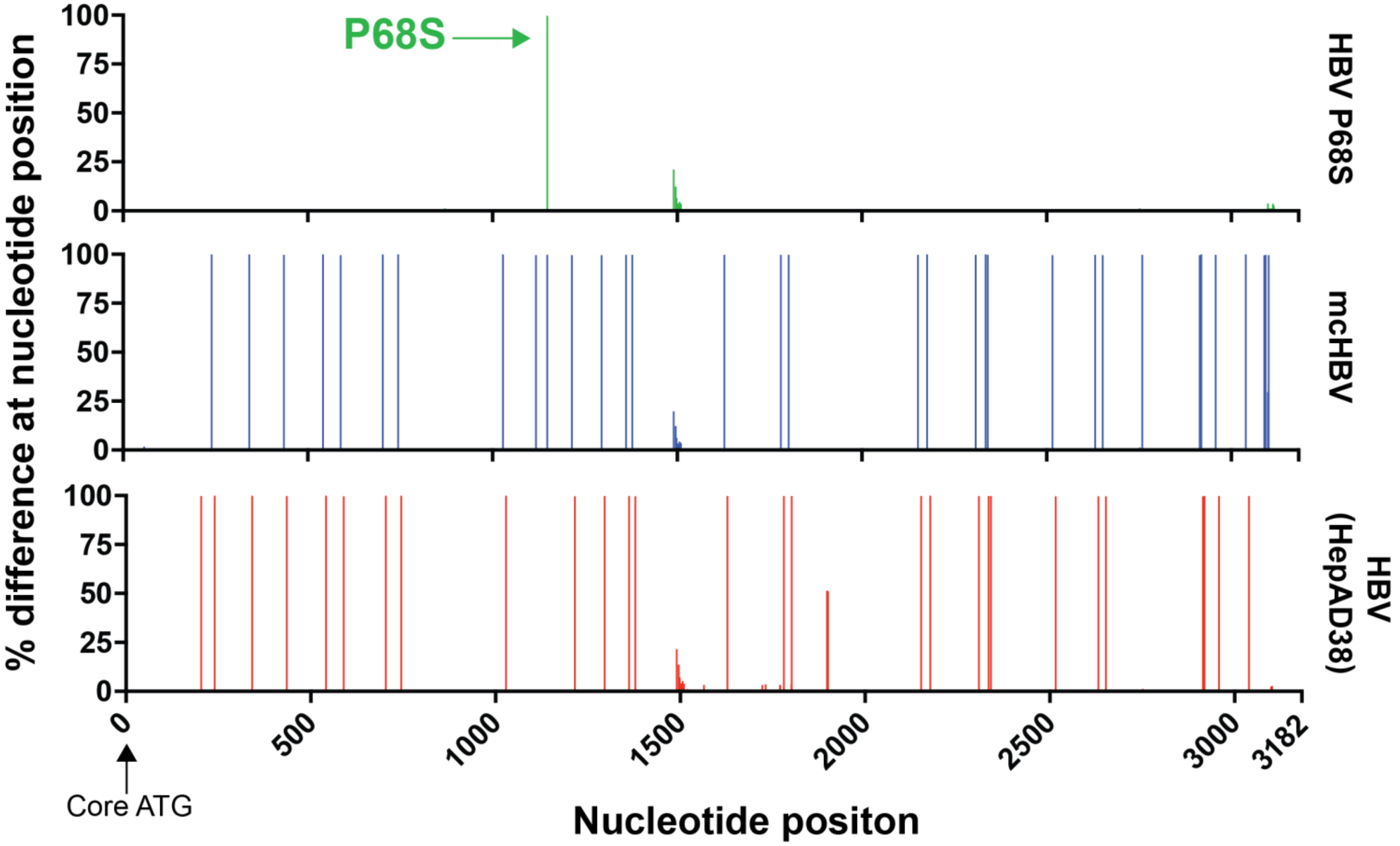

Supplementary Figure 1. Deep sequencing of HBV stocks. HBV stocks were PCR amplified using overlapping amplicons and deep sequenced using an Illumina MiSeq. Reads were aligned against the HBV genotype D serotype ayw described in Pasek *et al.*[26]. Colored spikes represent the fraction of reads that differ from the reference at each nucleotide position. Nucleotides are numbered beginning with the adenine of the HBV core ATG start codon.

Supplementary Figure 2.

Rhesus macaque PH supernatant - HDAd (10x dilution) **Rhesus macaque PH supernatant - AAV8 (10x dilution)**

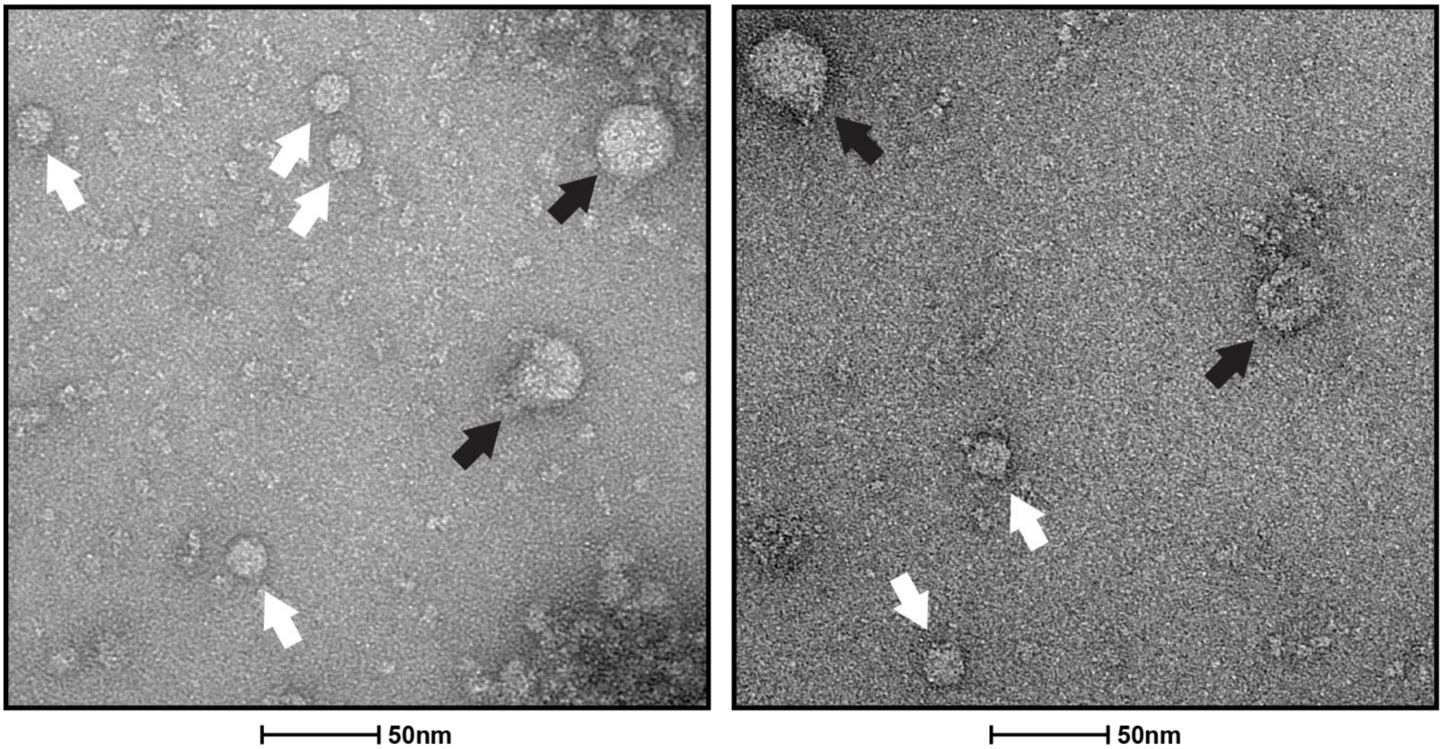

Supplementary Figure 2. Electron microscopy of Dane particles secreted from HBV-infected rhesus macaque PH. Left - Rhesus macaque PH treated with HDAd-hNTCP. Right - Rhesus macaque PH treated with AAV8-hNTCP. Black arrow = Dane particle. White arrow = Spherical bodies consistent with virion-like HBsAg particles lacking HBV DNA and core.

Supplementary Figure 3.

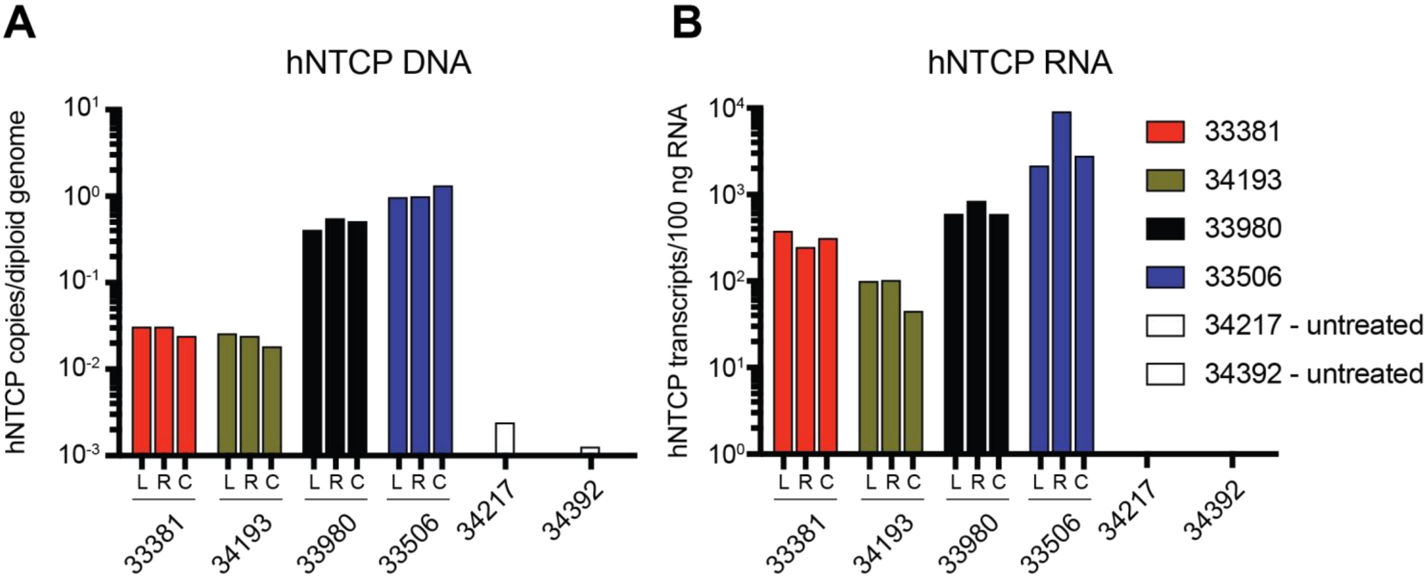

Supplementary Figure 3. Analysis of liver transduction and hNTCP expression. A) qPCR for hNTCP DNA shows low transduction levels as assessed by hNTCP DNA copies per diploid genome. B) qRT-PCR for hNTCP RNA shows expression of hNTCP in all animals in all liver lobes assessed. L = left liver lobe; R = right liver lobe; C = caudate liver lobe.

Supplementary Figure 4.

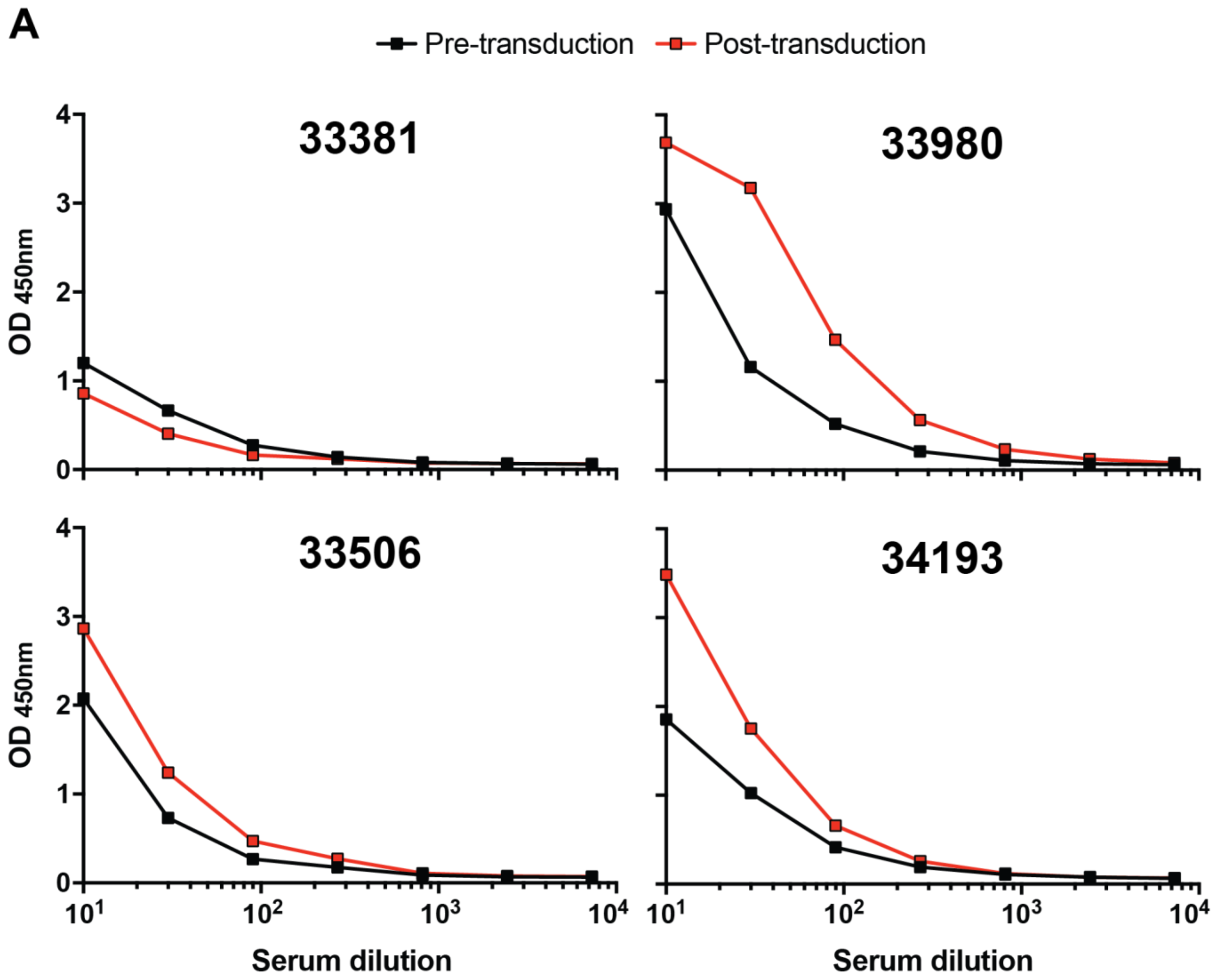

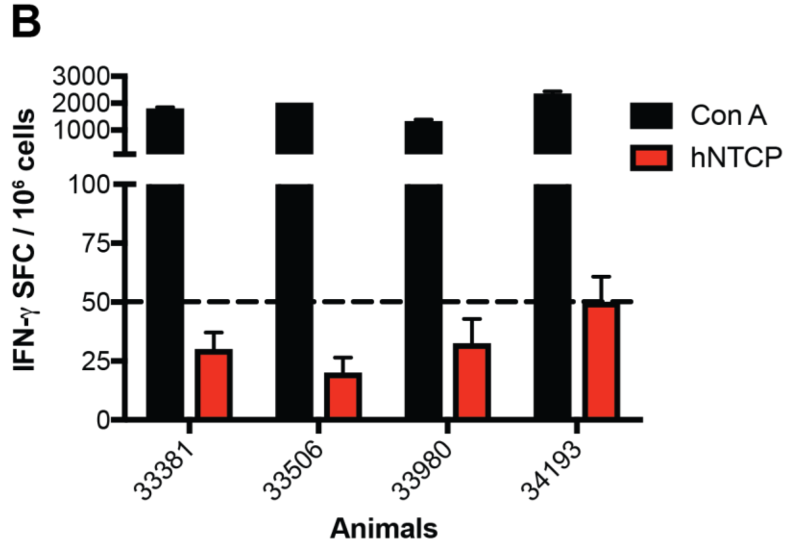

Supplementary Figure 4. Measurement of anti-hNTCP immune responses. A) Anti-hNTCP ELISA using HBV-infected rhesus macaque serum. Positive signal was defined as 3-fold increase in OD 450nm over pre-transduction at each serum dilution. No animals exhibited hNTCP specific humoral responses. B) ELISpot results show no cellular responses against recombinant hNTCP above assay limit of detection (50 IFN-γ SFC/10^6 cells). SFC = spot-forming cells. Con A = concanavalin A (positive control). Bars represent standard error of measurement from two ELISpot replicates.

Supplementary Figure 5.

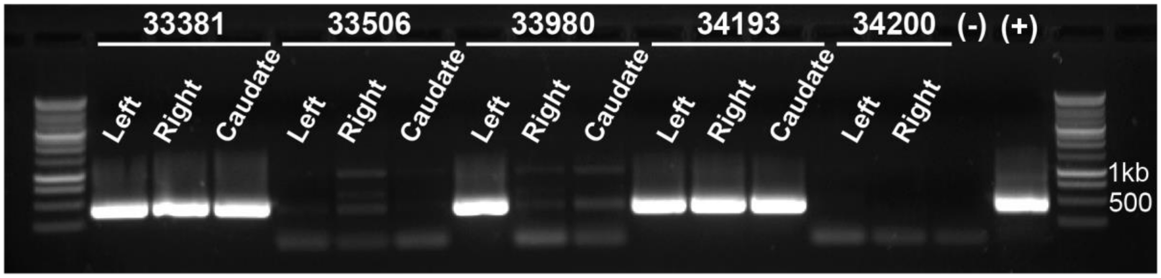

Supplementary Figure 5. Nested PCR shows presence of HBV DNA in liver sections obtained from HBV-infected rhesus macaques. (-) negative control with no template. (+) positive control with HBV DNA template extracted from high-titer stock of HBV.

Supplementary Table 1. Competitive anti-HBc ELISA of MCM cohort at ONPRC

ONPRC MCM ID	Optical Density (450nm)
32843	1.415
32844	1.507
32845	1.436
32846	0.615
32847	0.734
32848	0.747
32849	0.473
32850	1.077
32851	1.176
33450	0.925
33454	0.848
33455	1.425
33457	0.321
33460	1.403
33461	1.276
34067	0.688
34068	0.417
34661	1.317
34662	1.196
34663	1.234
34664	0.389
34665	1.200
34666	1.414
34668	1.276
34669	1.246
34670	1.073
34837	0.686
Negative (mean)	1.422
Positive (mean)	0.010
Cutoff	0.284

negative
positive

Supplementary Table 2. Competitive anti-HBc ELISA of HBV-challenged MCM at ONPRC

	Optical Density 450nm	
MCM	Day 0	Day 56
33451 (HBV P68S)	1.332	1.351
33453 (HBV P68S / CD3-IT)	0.993	1.024
34084 (mcHBV)	n.d.	1.107
34079 (mcHBV / CD3-IT)	1.432	1.484
Negative (mean)	1.680	
Positive (mean)	0.01	
Cutoff	0.336	

negative
positive

Supplementary Table 3. Competitive anti-HBc ELISA of HBV-challenged rhesus macaques at ONPRC

	Optical Density 450nm	
Rhesus macaque	Day 0	Day 56
33506 (AAV)	1.832	0.055
33980 (AAV)	1.488	1.434
33381 (HdAd)	1.291	1.517
34193 (HdAd)	1.341	1.377
Negative (mean)	1.680	
Positive (mean)	0.01	
Cutoff	0.336	

negative
positive

Supplementary Table 4. Serology markers in HBV-challenged rhesus macaques

Anti-HBe measurements (relative detection)

Sample	S/CO	Reactivity
33381	1.75	Nonreactive
34193	1.90	Nonreactive
33506	2.01	Nonreactive
33980	2.01	Nonreactive
Cutoff	≤1.00	-

Anti-HBs measurements (absolute detection of antibodies)

Sample	mIU/ml
33381	0.00
34193	0.00
33506	0.00
33980	0.00

HBsAg measurements (relative detection)

Sample	S/CO	Reactivity
33381	0.71	Nonreactive
34193	0.17	Nonreactive
33506	0.15	Nonreactive
33980	0.20	Nonreactive
Cutoff	≥1.00	-
